# Combinatorial intervention with dental pulp stem cells and sulfasalazine in a rat model of ulcerative colitis

**DOI:** 10.1007/s10787-024-01532-w

**Published:** 2024-07-29

**Authors:** Riham M. Aly, Rehab S. Abohashem, Hanaa H. Ahmed, Alyaa S. Abdel Halim

**Affiliations:** 1 Basic Dental Science Department, Oral & Dental Research Institute, National Research Centre, 33 El Bohouth St, Dokki, Giza, Egypt; 2 Stem Cells Lab, Center of Excellence for Advanced Sciences, National Research Centre, Dokki, Giza, Egypt; 3 Hormones Department, Medical Research and Clinical Studies Institute, National Research Centre, Dokki, Giza, Egypt; 4 Biochemistry Department, Faculty of Science, Ain Shams University, Cairo, Egypt

**Keywords:** Dental pulp stem cells, Sulfasalazine, Ulcerative colitis, Acetic acid, Proinflammatory, Oxidative stress

## Abstract

**Background:**

Ulcerative colitis is an inflammatory bowel disease (IBD) that involves inflammation of the colon lining and rectum. Although a definitive cure for IBD has not been identified, various therapeutic approaches have been proposed to mitigate the symptomatic presentation of this disease, primarily focusing on reducing inflammation. The aim of the present study was to evaluate the therapeutic potential of combining dental pulp stem cells (DPSCs) with sulfasalazine in an acetic acid-induced ulcerative colitis rat model and to assess the impact of this combination on the suppression of inflammatory cytokines and the regulation of oxidative stress in vivo.

**Methods:**

Ulcerative colitis was induced in rats through transrectal administration of 3% acetic acid. The therapeutic effect of combining DPSCs and sulfasalazine on UC was evaluated by measuring the colonic weight/length ratio and edema markers; performing histopathological investigations of colon tissue; performing immunohistochemical staining for NF-κB-P65 and IL-1β; and evaluating oxidative stress and antioxidant indices via ELISA. Moreover, the proinflammatory markers NF-κB-P65, TNF-α and TLR-4 were assessed in colon tissue via ELISA. Furthermore, qRT‒PCR was used to estimate the expression levels of the TLR-4, NF-κB-P65, and MYD88 genes in colon tissue.

**Results:**

The investigated macroscopic and microscopic signs of inflammation were markedly improved after the combined administration of sulfasalazine and DPSCs, where a noticeable improvement in histological structure, with an intact mucosal epithelium and mild inflammatory infiltration in the mucosa and submucosa, with slight hemorrhage. The administration of either DPSCs or sulfasalazine, either individually or in combination, significantly reduced ROS levels and significantly increased XOD activity. The immunohistochemical results demonstrated that the combined administration of DPSCs and sulfasalazine attenuated NFκB-p65 and IL-1β expression. Finally, the combined administration of DPSCs and sulfasalazine significantly downregulated MyD88, NF-κB and TLR4 gene expression.

**Conclusions:**

Cotreatment with DPSCs and sulfasalazine had synergistic effects on ulcerative colitis, and these effects were relieved.

## Introduction

Ulcerative colitis (UC) is a chronic inflammatory condition that predominantly affects colon and rectal tissues. The global incidence of ulcerative colitis is expected to reach 5 million cases by 2023, with an increasing occurrence observed worldwide. Environmental and genetic susceptibility are regarded as the primary predisposing factors for the development of UC (Le Berre et al. 2023).

The cause and development of ulcerative colitis (UC) are multifaceted. Ulcerative colitis (UC) is thought to be caused by a disruption in the balance between the microbiota in the intestines and the immune system of the intestinal lining, leading to an excessive amount of inflammation in the intestines. Consequently, an imbalance in the composition of the gut microbiota plays a role in the development of ulcerative colitis (Ağagündüz et al. 2023b; Kalkan et al. 2023). Recent research focused on alterations in gut microbiota within various levels of the intestine (Shen et al. 2018). Changes mostly take place in the outer mucus layer of the colon, highlighting its importance in comprehending the characteristics of gut flora in inflammatory bowel disease (IBD) and the advancement of the disease. A number of studies indicate that probiotics may have a preventive effect on UC recurrence (Ağagündüz et al. 2022, 2023c, a; Açar et al. 2023). Although probiotics alone may not be effective in reaching UC recovery, a combination of antibiotics and probiotics has demonstrated potential in managing UC complications (Dhillon and Singh 2020).

While a definitive cure for UC has not been identified, various therapeutic approaches have been proposed to mitigate the symptomatic presentation of this disease, primarily focusing on reducing inflammation. The first line of treatment is referred to as “symptomatic relief”. Patients are often prescribed drugs such as aminosalicylate derivatives, including sulfasalazine, as part of this strategy to reduce acute inflammation in the gastrointestinal tract. Despite their side effects, corticosteroids are also prescribed to patients to suppress the production of proinflammatory cytokines by modulating the patients’ immune response. Nevertheless, both approaches are regarded as temporary solutions and fail to address the long-term complexities or repair the mucosal harm induced by the disease (Khan et al. 2019; Sugihara and Kamada 2021).

On the other hand, unconventional strategies such as stem cell-based therapies are rapidly emerging as potential alternatives for the treatment of a wide range of diseases (Aly 2020). The proposed therapeutic approaches involving stem cells in many inflammatory diseases usually rely on their ability to modulate the immune response and their diminished immunogenicity. Mesenchymal stem cells (MSCs) are generally defined as a safe source of stem cells (Kolkundkar 2014; Robert 2014; Volarevic et al. 2018; Xie et al. 2020; Takashima et al. 2021). These cells are multipotent and can differentiate into various lineages. They also exhibit substantial immunomodulatory and anti-inflammatory effects (Abo El-Dahab et al. 2024).

Due to their immunoregulatory and pro-survival properties, mesenchymal stem cells therapy has emerged as a promising therapeutic approach for the treatment of inflammatory bowel disease (IBD). Mesenchymal stem cells (MSCs) mitigate disrupted inflammatory responses by releasing a wide range of anti-inflammatory substances (Saadh et al. 2023). The clinical effectiveness of therapy depends on the significant release of different secretory molecules from MSCs through paracrine systems. These mechanisms are necessary for controlling the gut immuno-microbiota and promoting the growth and specialization of nearby cells such as intestinal epithelial cells and stem cells. Chen et al. showed that injecting MSCs intravenously greatly decreased the severity of UC in a murine model and further improved survival (Chen et al. 2013). Their results demonstrated that MSCs successfully entered the colon that had been affected by injury, and effectively promoted the growth of intestinal epithelial cells in addition to the differentiation of intestinal stem cells.

Several clinical trials have thoroughly studied the safety and effectiveness of MSCs therapy in patients with inflammatory bowel disease (Ko et al. 2021; Lopez-Santalla and Garin 2021; Vieujean et al. 2021; Saadh et al. 2023). Specifically, MSCs derived from bone marrow and adipose tissue, have been utilized in several clinical trials for IBD therapy either through local injections or intravenous infusions. Vladimirovich and Asfold investigated the safety and effectiveness of mesenchymal stem cells as a therapy for inflammatory bowel disease (IBD). They reported that the injection of local MSCs displayed long-term effectiveness, accompanied by a high level of safety (Vladimirovich and Asfold Ivanovich 2015). Additionally, Knyazev et al. 2015 investigated the long-term (5 years) effects on the safety and effectiveness of bone marrow-derived mesenchymal stem cells and standard immunosuppressive agents in patients suffering from Crohn’s disease clinically (Knyazev Oleg et al. 2015). The study found no significant differences in the occurrence of toxicity, infections or worsening of the inflammatory conditions. Therefore, cell-based therapy was deemed to be safe for implementation in clinical settings. The most frequently used routes for cell administration were intravenous and intracolonic approaches with the typical dosage administered in the majority of investigations being approximately 1–2 × 10^6^ cells per kilogram.

While the short-term safety and practicality of MSC transplantation have been confirmed, some limitations need to be addressed before they may be utilized in clinical settings (Farge et al. 2022; Gabriel et al. 2023). Bone marrow stem cells have been the predominant cell source, followed by umbilical cord and adipose tissue-derived stem cells (Welling et al. 2011; Zhang et al. 2018; Dige et al. 2019), and MSCs from sources that are easier to obtain like dental-derived stem cells have not been thoroughly investigated.

Dental-derived stem cells are a type of MSC that exhibit a significant degree of multipotency owing to their neural crest origin. This origin enables these cells to demonstrate a higher level of versatility in terms of their capacity to differentiate into various cell lineages. Therefore, when clinical application is considered, dental pulp stem cells (DPSCs) are often prioritized due to their ease of access, lack of ethical controversy and reduced risk of immune rejection. Moreover, DPSCs can successfully home to sites of tissue damage, reduce inflammation, and modulate host immune reactions (Che et al. 2022; Shi et al. 2022).

We hypothesized that combining DPSCs with a widely prescribed medication such as sulfasalazine at low concentrations could synergistically harness the benefits of both treatment strategies. Therefore, the objective of the present research was to examine the therapeutic outcomes of the combination of DPSCs with sulfasalazine in an acetic acid-induced ulcerative colitis rat model and to evaluate the effectiveness of this cotreatment in restraining inflammatory responses and prohibiting oxidative processes in vivo. Establishing the advantageous efficacy of this approach in an animal model of ulcerative colitis will help to confirm its use as a potential therapeutic alternative in subsequent clinical trials.

## Materials and methods

### Phase I (in vitro study)

#### (1) Isolation of dental pulp mesenchymal stem cells

##### Chemicals and reagents

Dulbecco’s modified Eagle’s medium (DMEM), fetal bovine serum (FBS), antibiotics, including streptomycin and penicillin, and phosphate-buffered saline (PBS) were purchased from Bio West, France. All the other chemicals were of analytical grade.

*(a) Dental pulp tissue collection and dental pulp stem cell isolation. *The experimental protocol of this research was reviewed by the Ethical Committee of Medical Research of the National Research Centre, Egypt, and was granted approval (2,434,052,023). Healthy human impacted third molars (*n* = 3) were obtained from healthy individuals aged 18 to 24 years who were undergoing extraction of impacted third molars. After providing informed consent, the human dental pulp stem cell (DPSC) isolation protocol followed that of Gronthos et al. (Gronthos et al. 2000), with some modifications (Ahmed et al. 2021; Aly et al. 2022). Briefly, the extracted molars were transferred to Dulbecco’s modified Eagle’s medium (DMEM) supplemented with antibiotics (100 U/ml penicillin and 100 mg/ml streptomycin). The molars were split open, and the pulp tissue was gently excavated and rinsed using phosphate-buffered saline (PBS) supplemented with penicillin and streptomycin. The dental pulp tissue was cut into tiny pieces and subsequently subjected to 2 mg/ml collagenase type I (Serva Electrophorese, Germany) for 15 min at 37 °C with constant shaking. Single-cell suspensions were cultured with DMEM (Lonza), 15% fetal bovine serum (Lonza), and 10,000 U/ml penicillin and 10,000 ng/ml streptomycin and then incubated in a humidified atmosphere of 5% CO_2_ at 37 °C. The cells were regularly checked with a phase contrast inverted microscope, and the culture medium was replaced every other day. The cells were passaged when 70% confluence was achieved by TrypleSelect. Cells of the third and fourth passages were utilized in the subsequent experiments.

*(b) Flow cytometric surface marker expression analysis for characterization of isolated DPSCs. *Flow cytometric analysis (FACS) for CD90, CD105, CD45, and CD34 was performed using a Cytomics FC500 flow cytometer (Beckman Coulter, USA) to confirm the presence of MSCs within the isolated cells. The subsequent procedures were implemented to quantify the expression of MSC markers using CXP software version 2.2 (Beckman Coulter, USA). A total of 1106 cells/ml were incubated at 4 °C in the dark with 10 ml of monoclonal antibodies against CD34 and CD45 to rule out hematopoietic origin, as well as CD90 and CD105 to confirm mesenchymal stem cell identity (Beckman Coulter, USA (Hanna et al. 2023, 2024). Isotypes served as the control group. Following a 20-min period of incubation, tubes containing the monoclonal-treated cells were supplied with 2 ml of PBS containing 2% FBS. After centrifugation for 5 min at 2500 rpm, the supernatant was discarded, and the cells were resuspended in 500 ml of PBS supplemented with 2% FBS.

### Phase II (in vivo study)

#### Chemicals and reagents

Acetic acid was purchased from Sigma-Aldrich (USA). Sulfasalazine was acquired from Academia International Trading.

#### Animal selection

All animals received humane care and handling according to the guidelines of the Medical Ethical Committee of the National Research Centre (NRC), Egypt, as well as the Institutional Animal Care and Use Committees (IACUCs) guidelines and recommendations. Ethical approval for animal testing was granted by the Medical Ethical Committee of the National Research Centre (Acceptance no. 2434052023).

A total of 50 mature male Wistar rats weighing between 180 and 220 g were acquired from the Animal House Colony of the National Research Centre (NRC), Egypt. The animals were provided unrestricted access to commercially available standard pellet food and fresh potable water throughout the entire trial period. Following a 2-week period of adjustment, the animals were placed in polypropylene cages in a room with a controlled temperature (25 ± 1 °C) and artificial lighting (12-h dark/light cycle). The space was kept free from any potential chemical contamination.

#### Induction of colitis

To induce colitis, rats were intrarectally injected with a single dose of 1 ml of 3% acetic acid following the methods of Fawzy et al. (2021). The control subjects were intrarectally injected with 1 ml of saline.

#### Experimental design and animal grouping

The rats were divided into five groups (*n* = 5):

Group (1): A normal control (NC) in which the rats were injected intrarectally with a single dose of 1 ml of saline. After 24 h, the rats received an intraperitoneal single dose of 1 ml of saline and were orally administered 1 ml of saline for 7 days.

Group (2): A positive control (PC) (ulcerative colitis) in which the rats were intrarectally administered a single dose of 1 ml (3% acetic acid) according to the modified method of Fawzy et al. (2021).

Group (3): Ulcerative colitis + dental pulp mesenchymal stem cells (DPSCs), in which the rats were intrarectally administered a single dose of 1 ml of 3% acetic acid; after 24 h, the rats received a single dose of an intraperitoneal injection of 2X10^6^ DPSCs in 1 ml of PBS (Yousefi-Ahmadipour et al. 2019).

Group (4): Ulcerative colitis + DPSCs + sulfasalazine, in which the rats were intrarectally administered a single dose of 1 ml (3% acetic acid), and after 24 h, the rats received a single dose of intraperitoneal injection of 2 × 10^6^ DPSCs; similarly, the rats were administered sulfasalazine at a dose of 30 mg/kg b.wt. dissolved in 1 ml of saline orally for 7 days.

Group (5): Ulcerative colitis + sulfasalazine, in which the rats were intrarectally administered a single dose of 1 ml (3% acetic acid), and after 24 h, the rats were administered sulfasalazine at a dose of 30 mg/kg b.wt. dissolved in 1 ml of saline orally for 7 days (Soliman et al. 2016).

All the rats were decapitated 15 days after the start of the experiment.

#### (5) Colon tissue collection

Upon the completion of the treatments, the animals were euthanized via cervical dislocation. Subsequently, the colon was quickly removed and split into three sections. The first section was homogenized in ice-cold PBS (pH 7.4). The homogenates (10% w/v) were then centrifuged for 15 min at 4 °C and 4000 rpm to obtain the supernatants, which were subsequently collected and stored at −20 °C until biochemical analysis. The second section was frozen in liquid nitrogen immediately and stored at − 80 °C prior to molecular genetic analysis. For histological and immunohistochemical procedures, the third section was fixed in 10% formalin saline.

### Phase 3 (analysis and examination)

#### (1) Macroscopic assessments of colon tissues and edema marker determination

Rats were killed by cervical dislocation under anesthesia, and the colon tissues were excised.

For macroscopic evaluation, the excised colons were laid out on a white surface and photographed. The weight and length of each colon were measured, and the edema marker was determined using the equation: edema marker = colon weight (g) × colon length (cm) × 100 (Wang et al. 2022).

#### (2) Biochemical analysis

##### (a) Assessment of proinflammatory marker levels via enzyme-linked immunosorbent assays (ELISAs)

Nuclear factor kappa B (NF-κB- P65), tumor necrosis factor alpha (TNF-α), and Toll-like receptor 4 (TLR-4) concentrations in colon tissue homogenates were measured using ELISA kits (ELK Biotechnology, Wuhan, China) according to the manufacturer’s instructions.

##### (b) Evaluation of oxidative stress indices

Xanthine oxidase (XOD) and reactive oxygen species (ROS) were measured in colon homogenates by using ELISA kits acquired from MyBioSource (USA).

#### (3) Histopathological and immunohistochemical examination of colon tissues

Following a 24-h fixation of the colon tissues in 10% neutral formalin saline, the tissues were rinsed in water, and serially diluted alcohol (methyl, ethyl, and 100% ethyl) was used to dehydrate them. The specimens were cleared in xylene and embedded in paraffin bee wax at 56 °C in a hot air oven for 24 h. A rotary microtome was used to segment paraffin bee wax tissue blocks at a thickness of 4 microns. After routine dewaxing and hydration, hematoxylin and eosin (H&E) staining was applied, and an optical microscope was used to detect histological changes in the colon tissues (Drury 1983).

For immunohistochemical analysis, paraffin sections were mounted on positively charged slides by using the avidin biotin‒peroxidase complex (ABC) method. A NF-κB-p65 (rabbit) polyclonal antibody (Elabscience, Cat# E-AB-32232, Dil.: 1:100) and rabbit anti-IL-1β polyclonal antibody (rabbit) (Novusbio, Cat# NBP1-19775SS, Dil.: 1:100) were used. Sections from each group were exposed to these antibodies, followed by the addition of the necessary reagents for the ABC technique (Vectastain ABC-HRP kit, Vector Labs). The marker expression was tagged using peroxidase and stained with diaminobenzidine (DAB), a product manufactured by Sigma, to identify the antigen–antibody complex. Nonimmune serum was used as a negative control instead of the primary or secondary antibody. The IHC-stained sections were analyzed using an Olympus microscope (model BX-53).

#### (4) Gene expression analysis by quantitative reverse transcriptase polymerase chain reaction (qRT–PCR)

Using the RNeasy Mini Kit from Qiagen, total RNA was extracted from colon tissues. One microgram of total RNA was reverse transcribed using a QuantiTect Reverse Transcription Kit (Qiagen). qPCR was carried out in accordance with the manufacturer's instructions using the Quantinova SYBR Green PCR Kit (Qiagen). The specific primers used for NF-κB, Myd88, TLR-4 and the housekeeping gene GAPDH were used for qPCR. qPCR was carried out under the following conditions: initial denaturation at 94 °C for 4 min; 40 cycles of 94 °C for 15 s, annealing at 60 °C for 20 s, and extension at 72 °C for 20 s; and a final extension step for 10 min at 72 °C. The reaction mixture had a total volume of 20 μl (12.5 μl of SYBR Premix Ex, 0.5 μl of each primer, 2 μl of cDNA and 4.5 μl of nuclease-free H_2_O). The sequences of primers used for each target gene are listed in Table 1. The main equations used were as follows: ∆Ct = Ct (gene of interest) – Ct (housekeeping gene) followed by ∆∆Ct = ∆Ct (treated sample) – ∆Ct (untreated sample). The overall formula for calculating the relative fold change (Li et al. 2012) was 2–∆∆C.

**Table 1:** Primer sequences for real-time quantitative reverse transcription-polymerase chain reaction (RT-qPCR)

Gene name	Forward primer	Reverse primer
NF-κB	CATGAAGAGAAGACACTGACCATGGAAA	TGGATAGAGGCTAAGTGT AGACACG
TLR-4	TGGCATCATCTTCATTGTCC	CAGAGCATTGTCCTCCCACT
Myd 88	GAGATCCGCGAGTTTGAGAC	TTGTCTGTGGGACACTGCTC
GAPDH	CACCCTGTTGCTGTAGCCATATTC	GACATCAAGAAGGTGGTGAAGCAG

### Statistical analysis

All the results are expressed as the mean ± SD. The data were analyzed by one-way analysis of variance (ANOVA) using the Statistical Package for the Social Sciences (SPSS) program (SPSS, Inc., Chicago, IL, USA), version 22, followed by the least significant difference (LSD) test to determine the significance of the difference between groups. Differences were considered significant when the *p* value was < 0.05.

## Results

### (1) Dental pulp stem cell characterization

**Fig.1:**
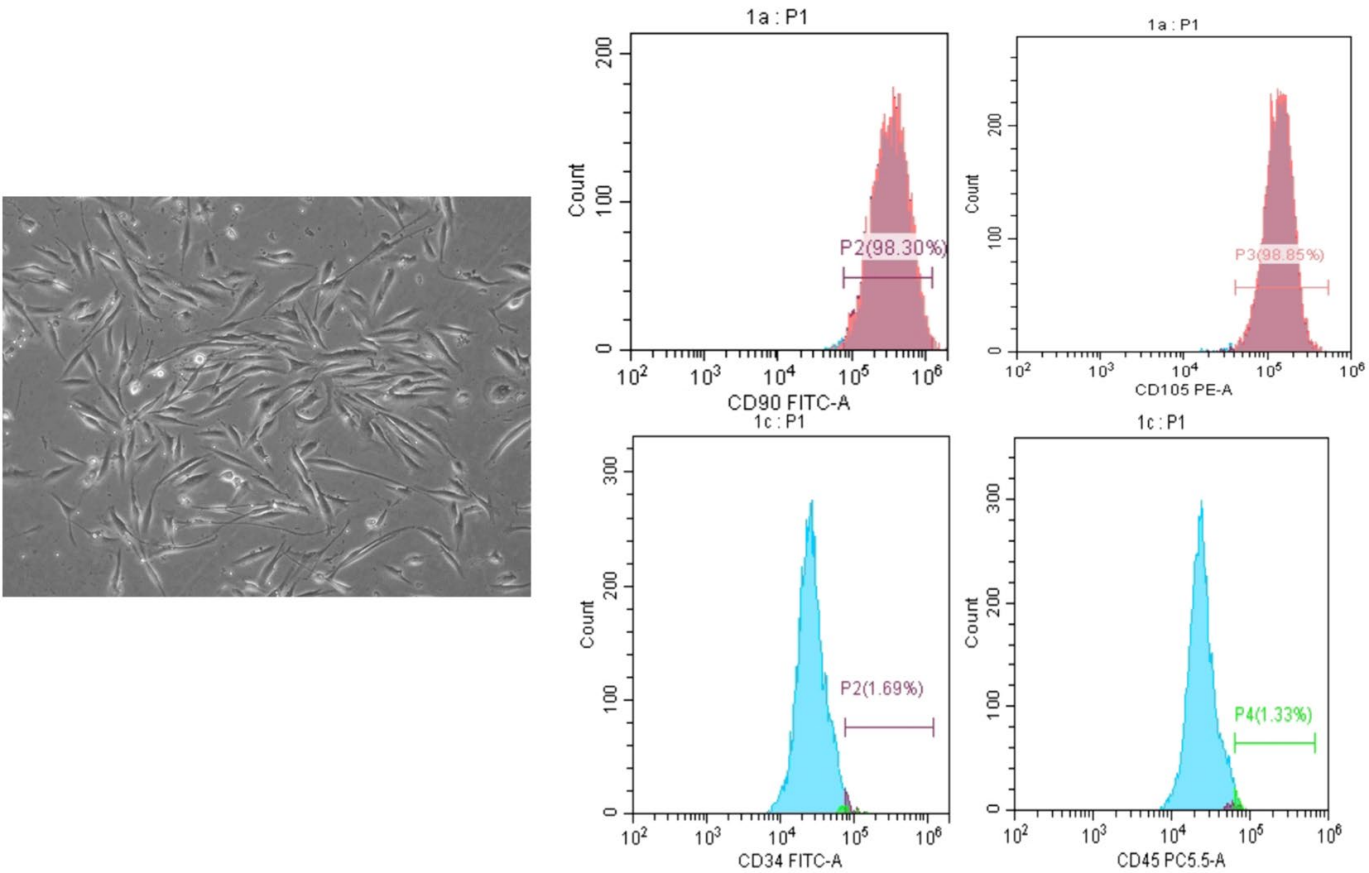
Morphological observation and flow cytometric characterization of isolated DPSCs*.* The cells exhibited a fibroblast-like appearance. The cells exhibited positive expression of CD90 (98.3%) and CD105 (98.8%) and negative expression of CD34 (1.69 Images of colon tissue 15 days %) and CD45 (1.33%)

A few days after culture, microscopic observation of the isolated cells revealed stellate-shaped fibroblast-like cells that were adherent to the culture plate and demonstrated signs of proliferation. Moreover, flow cytometric analysis confirmed that the isolated dental pulp stem cells (DPSCs) were mesenchymal stem cells (Fig. 1a). The isolated cells exhibited positive expression of specific mesenchymal stem cell markers, namely, CD90 and CD105, while the expression of the hematopoietic markers CD34 and CD45 was negative (Fig. 1b).

### (2) Combinatorial intervention with DPSCs and sulfasalazine mitigated the macroscopic appearance of the colon in ulcerative colitis-challenged rats

**Fig. 2:**
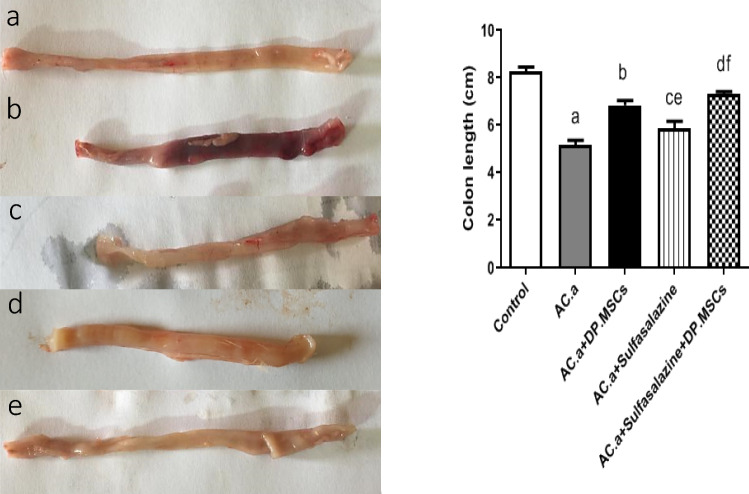
after ulcerative colitis induction (left): *(a) control, (b) 3% acetic acid, (c) DPSCs, (d) sulfasalazine 30, and (d) DPSCs* + *sulfasalazine 30*. Effect of different treatments on colon length in an induced ulcerative colitis rat model (right). a: Significant difference between the control and (AC treatments. a) group. b: Significant difference between (AC. a) and (AC. a) + DPSCs. c: Significant difference between (ACs. a) and (AC. a) + sulfasalazine. d: Significant difference between (AC. a) and (AC. a) + sulfasalazine + DPSCs. e: Significant difference between (ACs. a) + DPSCs and (AC. a) + sulfasalazine + DPSCs. f: Significant difference between (ACs. a) + sulfasalazine and (AC. a) + DPSCs. (AC. a): acetic acid, DP. MSCs: dental pulp mesenchymal stem cells. Significant difference at *P* < 0.05

The potential impact of coadministering DPSCs with sulfasalazine on mitigating inflammatory symptoms in ulcerative colitis was initially assessed by macroscopically observing the length of the colon in an ulcerative colitis rat model after 15 days of induction **(**Fig. 2a). The macroscopic appearance of the colon was significantly greater in the DPSC-treated rats than in the sulfasalazine-treated rats. A significant difference between acetic acid (AC. a) and acetic acid + sulfasalazine + DPSC, and acetic acid (AC. a) + sulfasalazine and acetic acid (AC. a) + DPSCs was evident. Notably, compared with those in all the other groups, the colon length in the DPSCs and sulfasalazine-treated rats was significantly greater **(**Fig. 2b).

### (3) Combined administration of DPSCs and sulfasalazine attenuated edema of colon tissue in ulcerative colitis-challenged rats

**Fig. 3:**
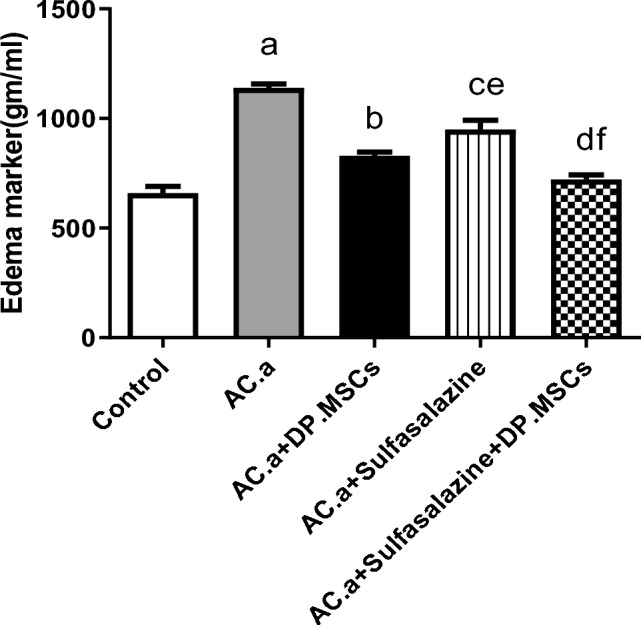
Effect of different treatments on edema marker of colon tissue of induced ulcerative colitis in rats. a: Significant difference between control and (AC. a) group. b: Significant difference between (AC. a) and (AC. a) + DPSCs. c: Significant difference between (AC. a) and (AC. a) + sulfasalazine. d: Significant difference between (AC. a) and (AC. a) + sulfasalazine + DPSCs. e: Significant difference between (AC. a) + DPSCs and (AC. a) + sulfasalazine + DPSCs. f: Significant difference between (AC. a) + sulfasalazine and (AC. a) + DPSCs. (*AC. a) *acetic acid*, DP. MSCs *Dental pulp mesenchymal stem cells*.* Significant difference at *P* < 0.05

The ratio of colon weight to length was investigated as a marker of ulcerative colitis-associated colonic edema. The edema marker demonstrated a significant increase in rats treated with acetic acid- (AC. a) induced ulcerative colitis compared to that in control counterparts. However, compared with that in the acetic acid- (AC. a) administered group, sulfasalazine administration alone significantly decreased edema markers in relation to acetic acid- (AC. a) administered group. Moreover, compared with acetic acid (AC. a) and sulfasalazine-treated groups. These results suggested that DPSC administration alone or in combination with sulfasalazine significantly attenuated colonic edema in acetic acid-induced ulcerative colitis in rats (Fig. 3).

### (4) DPSCs and sulfasalazine regulated reactive oxygen species (ROS) and xanthine oxidase (XOD) activity in induced ulcerative colitis-challenged rats

**Fig. 4:**
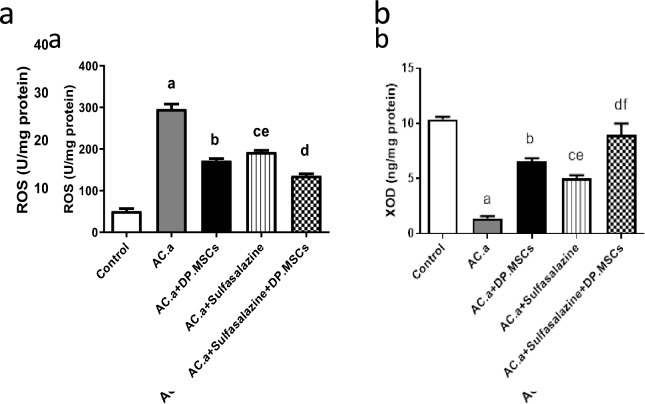
(**a**) Effect of different treatments on reactive oxygen species (ROS) of colon tissue of induced ulcerative colitis in rats. (**b**) Effect of different treatments on xanthine oxidase (XOD) activity of colon tissue of induced ulcerative colitis in rats. a: Significant difference between control and (AC .a) group. b: Significant difference between (AC. a) and (AC. a) + DPSCs. c: Significant difference between (AC .a) and (AC. a) + sulfasalazine. d: Significant difference between (AC. a) and (AC. a )+ sulfasalazine + DPSCs. e: Significant difference between (AC. a) + DPSCs and (AC. a) + sulfasalazine + DPSCs. f: Significant difference between (AC. a) + sulphasalazine and (AC. a) + DPSCs. Significant difference at *P* < 0.05. (*Ac. a) *acetic acid*, DP. MSCs *dental pulp mesenchymal stem cells

Any disruption in the equilibrium between antioxidation and oxidation is regarded as critical for determining the severity of ulcerative colitis. Initially, the level of ROS was assessed as an indicator of oxidative stress in the colon in all the groups under investigation (Fig. 4a). The level of ROS was markedly elevated in the groups with ulcerative colitis induced by acetic acid (AC. a) compared to the control group. Nevertheless, the administration of either DPSCs or sulfasalazine, either individually or in combination, led to a significant decrease in the level of reactive oxygen species (ROS) in contrast to that in the ulcerative colitis group induced by acetic acid (AC. a). The most significant suppression of ROS was observed when DPSCs were treated with sulfasalazine.. The antioxidant activity was also examined in the colonic tissue of all the groups under study. The administration of acetic acid (AC. a) treatment led to significant inhibition of XOD activity compared with that in the controls (Fig. 4b). Notably, the administration of DPSCs and sulfasalazine, either separately or in combination, significantly promoted XOD activity, contrary to that in the ulcerative colitis group induced by acetic acid (AC. a). The most prominent and pronounced increase in XOD was observed in the colons of rats with induced colitis when sulfasalazine and DPSCs were administered in combination.

### (5) DPSCs and sulfasalazine administration ameliorated histological alterations in colon tissues from induced ulcerative colitis rats

**Fig. 5:**
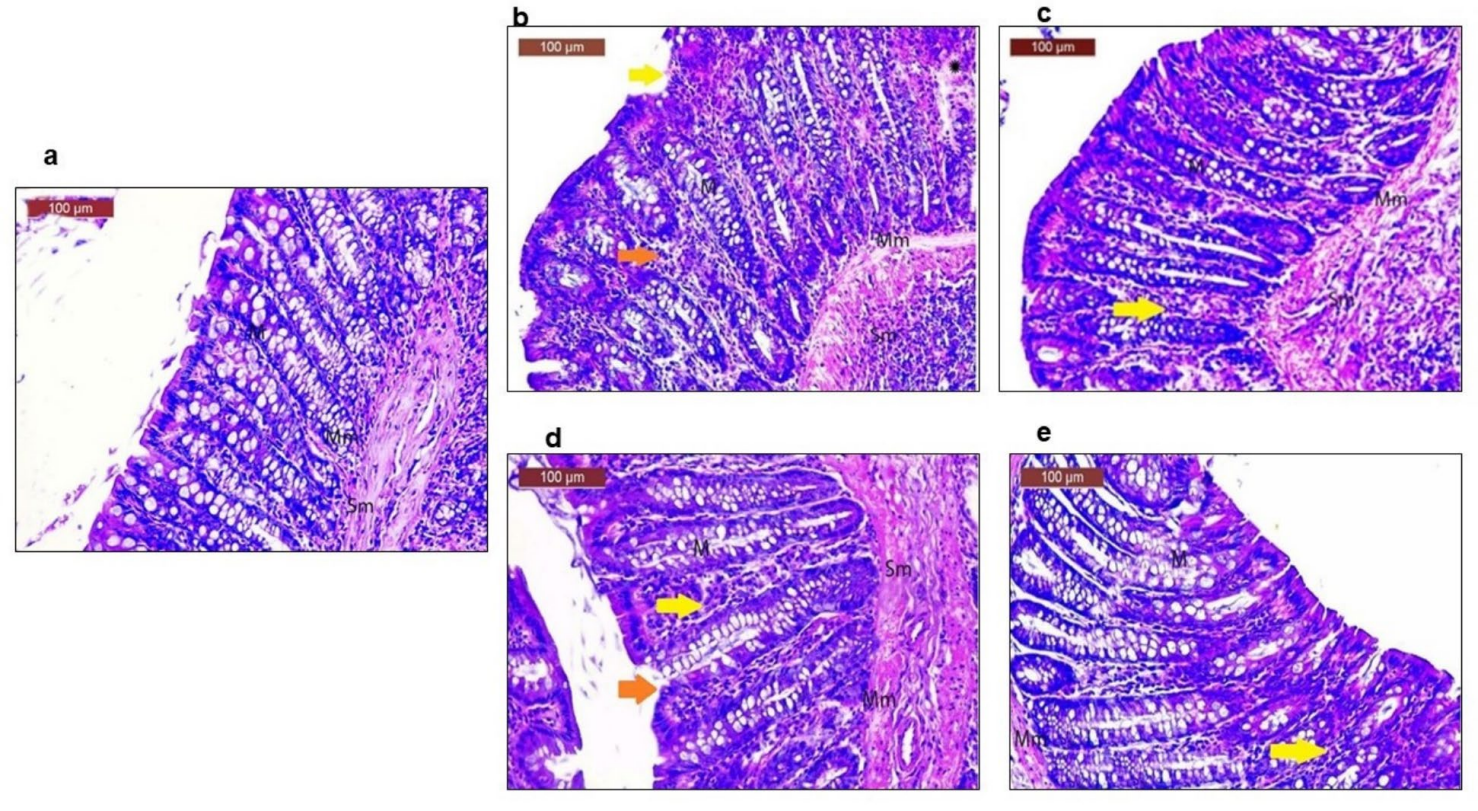
Representative photomicrographs of H&E-stained colon sections. (a) Photomicrograph of a section in the colon from the control group showing normal structure of mucosa with an intact epithelial surface (M), muscularis layers (Mm), and submucosa (Sm). (**b**) Groups treated with acetic acid demonstrating patchy loss of normal surface epithelium (arrowhead), distortion of normal crypts architecture, degenerated cells of mucosa (M), inflammatory infiltration in the mucosa (arrow) and submucosa (Sm) with the presence of hemorrhage (star). (**b**) Groups treated with acetic acid and hDPSCs showing normal histological structure with intact mucosal epithelium (M), muscularis layers (Mm) only slight inflammatory infiltration mucosa (yellow arrow) and submucosa (Sm). (**c**) Groups treated with acetic acid and sulfasalazine illustrating moderate improvement with minor loss of some surface columnar epithelial cells (arrowhead), there was mild inflammatory infiltration in the mucosa (arrow) and submucosa (Sm). (**d**) Groups treated with acetic acid and (sulfasalazine + hDPSCs) demonstrating noticeable improvement of the histological structure with intact mucosal epithelium (M), only a mild inflammatory infiltration in the mucosa (arrow), submucosa (Sm) and slight hemorrhage (star). *(a) Control, (b) (AC. a), (c) AC. a* + *DPSCs, (d) AC. a* + s*ulfasalazine, (e) (AC. a* )+ *sulfasalazine* + *DPSCs. Mucosa (M), submucosa (Sm), muscularis layers (Mm), slight hemorrhage (star)*

The control groups exhibited the typical histological characteristics of the mucosa, muscularis layers, and submucosa in the colon (Fig. 5** a**). The acetic acid (AC. a) group displayed patchy loss of the normal surface epithelium, distortion of the normal crypt architecture, degeneration of mucosal cells, and inflammatory infiltration in the mucosa and submucosa in the presence of hemorrhage (Fig. 5b). The group treated with acetic acid (AC. a) + DPSCs showed a normal histological structure with an intact mucosal epithelium and muscularis layers with only slight inflammatory infiltration of the mucosa and submucosa **(**Fig. 5c**)**. The group treated with a combination of acetic acid (AC. a) and sulfasalazine exhibited a modest improvement, accompanied by a slight loss of a few surface columnar epithelial cells. Additionally, slight inflammatory infiltration was observed in both the mucosa and submucosa **(**Fig. 5d**)**. In the group treated with acetic acid (AC. a) + sulfasalazine + DPSCs, the histological structure was noticeably improved, the mucosal epithelium was intact, and only mild inflammatory infiltration in the mucosa and submucosa was observed with slight hemorrhage **(**Fig. 5e**)**.

### (6) Administration of DPSCs and sulfasalazine downregulated the expression of IL-1β and NFκB-p65 in ulcerative colitis-challenged rats

**Fig.6:**
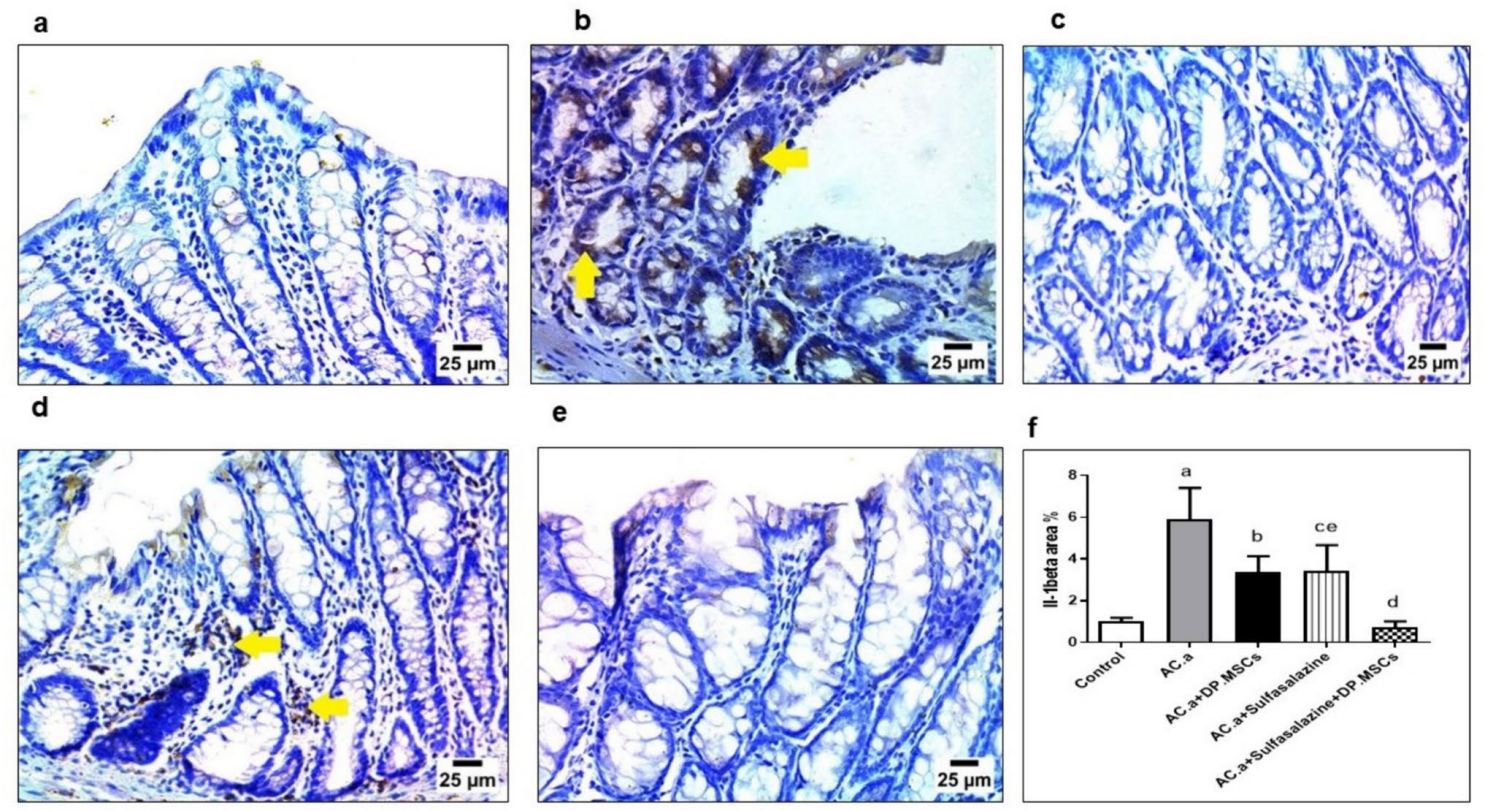
Immunohistochemical staining against IL-1β in colonic sections of different treatment groups. (**a**) Photomicrograph showing negative reaction for IL-1β in intestinal mucosa in the control group. (**b**) Groups treated with acetic acid showing strong positive reaction for IL1β in cytoplasm of intestinal glands (arrows). (**c**) Groups treated with acetic acid and hDPSCs showing negative reaction for IL-1β in the intestinal mucosa. (**d**) Groups treated with acetic acid and sulfasalazine illustrating strong positive reaction for IL1β in the cytoplasm of inflammatory cells in the intestinal mucosa (arrows). (**e**) Groups treated with acetic acid and (sulfasalazine + hDPSCs) showing negative reaction for IL-1β in the intestinal mucosa. (**f**) Expression of IL-1β in the studied groups. Data presented as mean and standard deviation. *(a) Control. (b) (AC. a). (c) (AC. a)* + *DPSCs. (d) (AC. )a* + s*ulfasalazine. (e) (AC. a)* + *sulfasalazine* + *DPSCs. Significant difference at P* < *0.05*

**Fig. 7:**
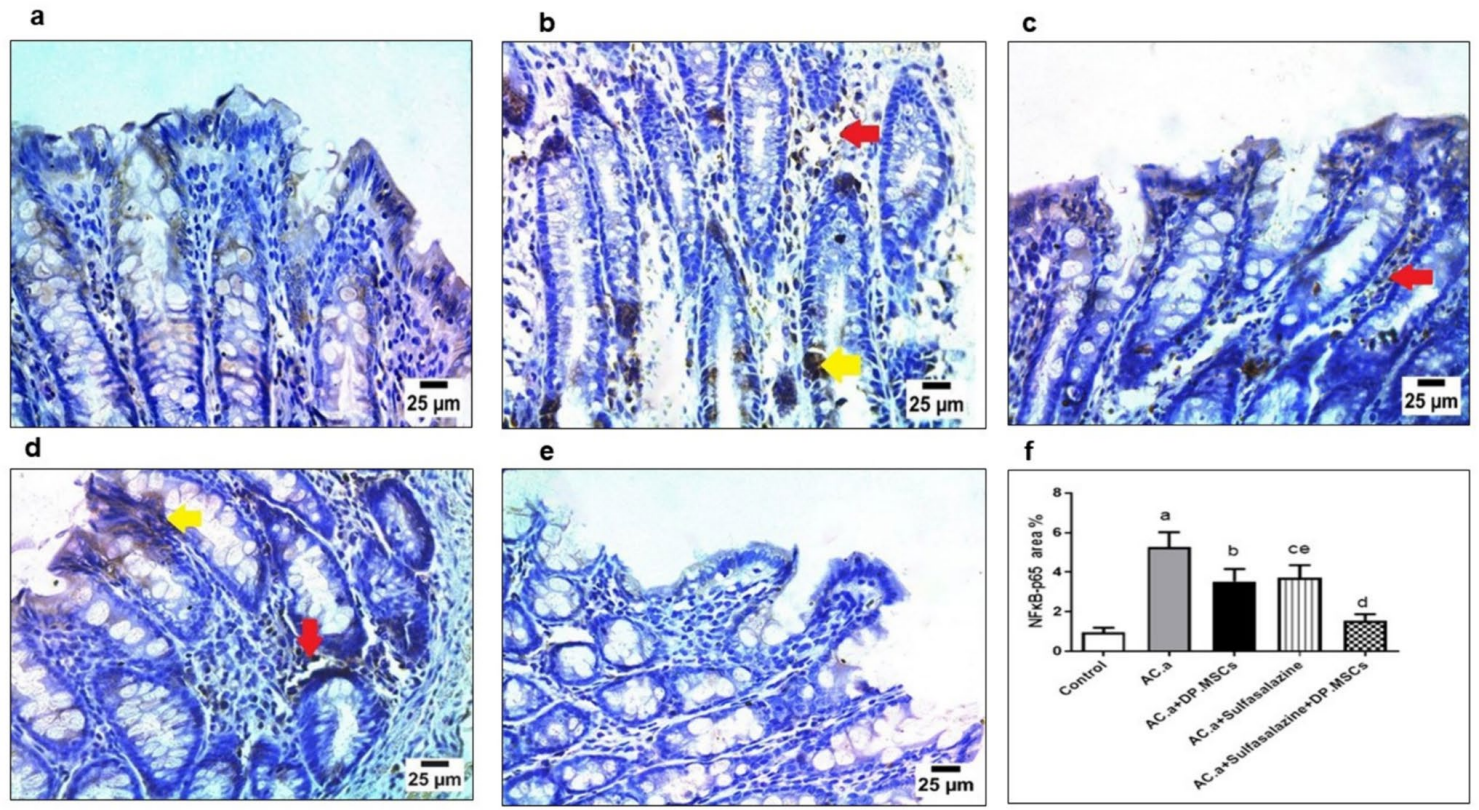
Immunohistochemical staining against NFκB-p65 in colonic sections of different treatment groups. (**a**) Photomicrograph showing negative reaction for NFKB-p65 in intestinal mucosa of the control group. (**b**) Groups treated with acetic acid showing strong positive reaction for NFKB-p65 in nuclei and cytoplasm of the intestinal epithelium (yellow arrow), in nuclei of inflammatory cells (red arrow) between intestinal villi (**c**) groups treated with acetic acid and hDPSCs showing positive reaction for NFKB-p65 in the nuclei of intestinal epithelium (red arrow). (**d**) Groups treated with acetic acid and sulfasalazine showing strong positive reaction for NFKB-p65 in the cytoplasm of intestinal villi (yellow arrow) and nuclei of inflammatory cells between villi (red arrow). (**e**) Groups treated with acetic acid and (sulfasalazine + hDPSCs) demonstrating negative reaction for NFKB-p65 in the intestinal mucosa. (f) Expression of NFKB-p65 in the studied groups. Data presented as mean and standard deviation. (**a**) Control. (**b**) (AC. a). (**c**) (AC. a) + DPSCs. (**d**) (AC. a) + sulfasalazine. (**e**) (AC. a) + sulfasalazine + DPSCs. Significant difference at *P* < 0.05

The expression of IL-1β and NFκB-p65 was investigated immunohistochemically to provide further insights into the inflammatory status of the colon tissue and the potential effectiveness of DPSC and sulfasalazine administration in modulating the inflammatory response. The expression of both IL-1β and NF-κB-p65 in the colon tissues of rats significantly increased in comparison to that of control rats when acetic acid (AC. a) was administered to induce ulcerative colitis in rats. Figures 6and 7 depict the percentage and intensity of positive cells stained for IL-1β and NF-κB-p65, respectively. Normal colon mucosa in the control group exhibited weak IL-1β staining on the cell membranes, which illustrated a staining intensity nearly equivalent to that of the background staining (Fig. 6a). Conversely, the acetic acid (AC. a) group cells exhibited a high percentage of IL-1β-positive cells, which were primarily localized in the nuclei, in comparison to the control group (Fig. 6b). Therefore, IL-1β expression greatly increased in response to the acetic acid (AC. a) group, whereas it markedly decreased in the groups that received DPSCs and sulfasalazine alone (Fig. 6c, d). Nevertheless, the expression levels of IL-1β were significantly lower in DPSCs administered in combination with sulfasalazine than in those in all the other groups (Fig. 6e). However, neither DPSCs nor sulfasalazine independently affected IL-1β expression compared to the control (Fig. 6f). NFκB-p65 was weakly expressed at the cell membrane of the colon mucosa in the control group (Fig. 7a). The acetic acid (AC. a) group demonstrated intense staining of the nuclei with a significantly increased number of positive cells (Fig. 7b). The DPSC- and sulfasalazine-treated groups showed significantly lower staining intensity and percentage of positive cells than did the acetic acid (AC. a) group (Fig. 7c). Remarkably, the combination of DPSCs and sulfasalazine significantly diminished both the staining intensity and percentage of cells positive for NF-κB-p65 compared to those of all the other groups (Fig. 7f). Like the findings observed with IL-1β staining, neither DPSCs nor sulfasalazine separately affected the expression of NF-κB-p65 compared to that in the control group (Fig. 7d, e). Taken together, these results demonstrated that the combined administration of DPSCs and sulfasalazine alleviated the expression of inflammatory markers, such as IL-1β and NF-κB-p65, indicating the potent role of their combined administration in the recovery of the inflammatory response induced by UC.

### (7) Administration of DPSCs and sulfasalazine suppressed the levels of proinflammatory cytokines in the colons of ulcerative colitis-challenged rats

**Fig. 8:**
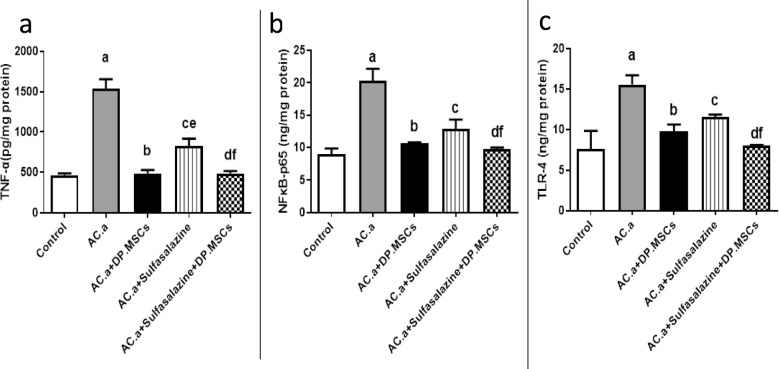
Effect of different treatments on the levels of proinflammatory cytokines according to ELISA. (**a**) TNF-α (pg/mg), (**b**) NFκB-p65 (ng/mg protein) and (**c**) TLR-4 (ng/mg protein). a: Significant difference between the control and (AC. a) group; b: significant difference between (AC. a) and (AC. a) + DPSCs; c: significant difference between (AC. a) and (AC. a) + sulfasalazine; d: significant difference between (AC. a) and (AC. a) + sulfasalazine + DPSCs; f: significant difference between (AC. a) + sulfasalazine and (AC. a) + DPSCs. A P value < 0.05 indicated a significant difference. *Ac. a *acetic acid*, DP. MSCs *dental pulp mesenchymal stem cells.

To investigate the improvement in the immune response that could have resulted from the administration of DPSCs alone or in combination with sulfasalazine, the levels of proinflammatory cytokines were analyzed in the colon tissues of all the subjects studied **(**Fig.8**)**. Enzyme-linked immunosorbent assays (ELISA) were utilized to assess the levels of TNF-α (Fig.8a), NF-κB-p65 (Fig.8b), and TLR-4 (Fig.8c) in the colon. Comparing acetic acid (AC. a) with those in the control group, the levels of TNF-α, NF-κB-p65, and TLR-4 in the colon were significantly greater. Although the administration of DPSCs or sulfasalazine alone resulted in a significant reduction in the levels of the tested proinflammatory cytokines in the colon, the combination of both DPSCs and sulfasalazine significantly resulted in an even greater reduction in the levels of those cytokines in the colon than the acetic acid (AC. a) group.

### (8) Administration of DPSCs and sulfasalazine downregulated the expression of the MyD88, NFκB and TLR-4 genes in the colon tissues of ulcerative colitis-challenged rats.

**Fig. 9:**
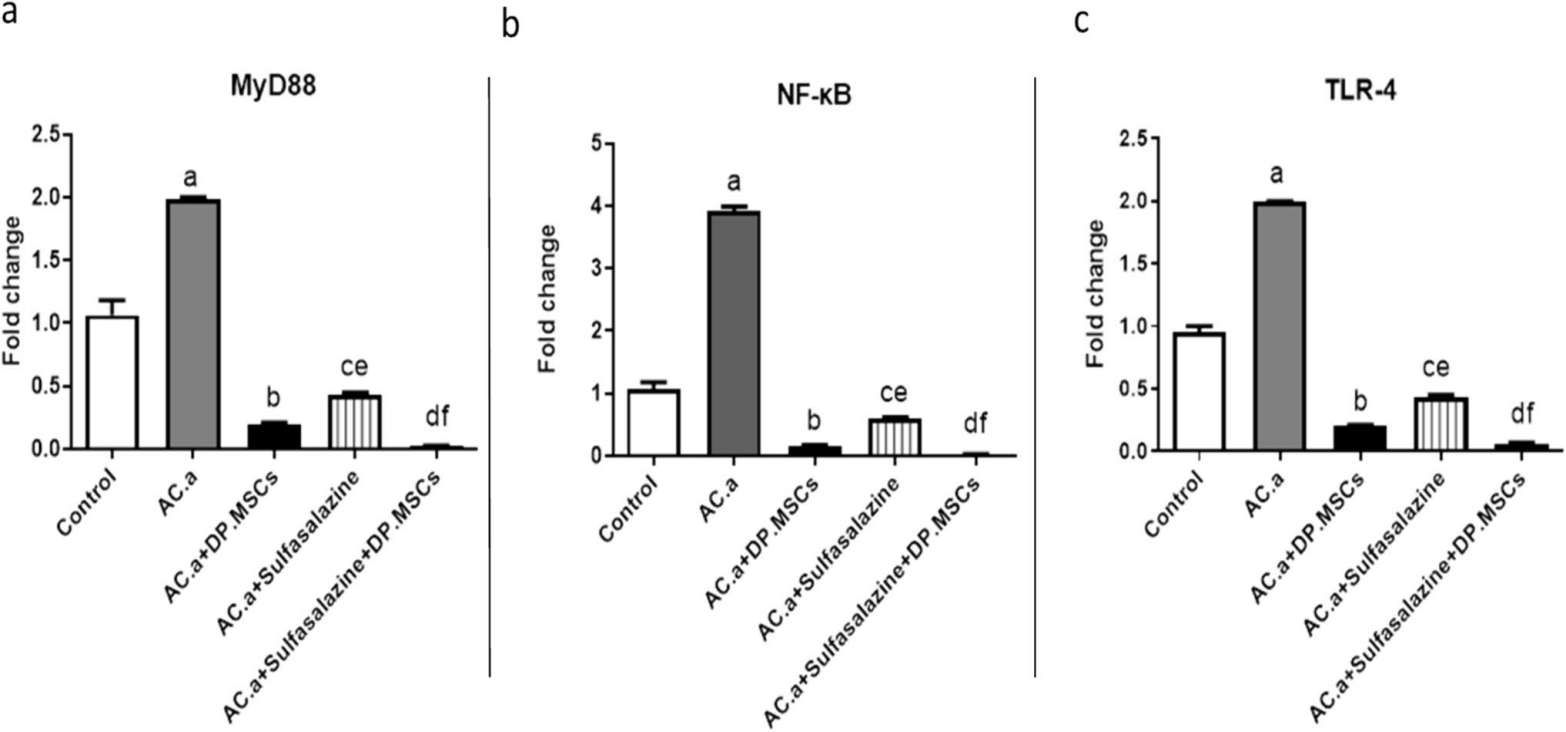
qRT-PCR of MyD88, NF-κB and TLR4 markers in colon tissue of ulcerative colitis-challenged rats. (**a**) Expression level of MyD88 gene. (**b**) Expression level of NF-κB gene (**c**) Expression level of TLR-4 gene. a: Significant difference between the control and (AC. a) group, b: Significant difference between (AC. a) and (AC. a) + DPSCs. c: Significant difference between (AC. a) and (AC. a) + sulfasalazine. d: Significant difference between (AC. a) and (AC. a) + sulfasalazine + DPSCs. e: Significant difference between (AC. a) + DPSCs and (AC. a) + sulfasalazine + DPSCs. f: Significant difference between (AC. a) + sulfasalazine and (AC. a) + DPSCs. Significant difference at *P* < 0.05. *Ac. a *acetic acid*, DP. MSCs *dental pulp mesenchymal stem cells

To validate the anti-inflammatory properties of DPSCs and sulfasalazine in inhibiting the progression of UC, gene expression analyses were conducted for MyD88, NF-κB and TLR-4 (Fig. 9). The expression levels of the MyD88, NF-κB and TLR-4 genes in rat colon tissues were significantly upregulated in the acetic acid (AC. a) group in comparison to the control counterparts. In contrast, the expression levels of the MyD88, NF-κB and TLR-4 genes in both the DPSC-treated group and the sulfasalazine-treated group were significantly lower than those in the acetic acid (AC. a) group. The combined administration of DPSCs and sulfasalazine significantly downregulated the MyD88 **(**Fig. 9a**)**, NF-κB (Fig. 9b) and TLR-4 (Fig. 9c) gene expression levels compared with those in the control group.

Ulcerative colitis is a chronic inflammatory disease of the intestine that unfortunately lacks a definitive therapeutic approach. During the initial phases, many patients are typically prescribed aminosalicylates, specifically sulfasalazine, which is the most frequently recommended anti-inflammatory medication. However, patients using sulfasalazine frequently experience severe side effects, including nausea, vomiting, and hemolysis. Thus, to mitigate these adverse effects, it is crucial to adopt innovative therapeutic strategies. One of these strategies is to integrate stem cells into the treatment regimen through combination therapy.

The purpose of the present research was to assess the efficacy of combining DPSCs with sulfasalazine in acetic acid-induced ulcerative colitis in rats. The proposed therapeutic effect of combining dental pulp stem cells (DPSCs) and sulfasalazine arises from the synergistic integration of the advantages offered by both approaches. DPSCs possess a potent immunomodulatory effect, which results in a reduction of autoimmune inflammation and facilitates the healing of the intestinal mucosa. The anti-inflammatory action of sulfasalazine enhances this effect. Consequently, this approach shall extend the length of time during which the disease is inactive, reduces the likelihood of recurring episodes, and lessens the frequency of being admitted to the hospital.

It has been previously documented that acetic acid administration in rats results in an ulcerative colitis model similar to that in humans (Fabia et al. 1992; Low et al. 2013; El-Akabawy and El-Sherif 2019). It causes damage to the distilled colon portion and results in nontransmural inflammation. Moreover, acetic acid also provokes necrotic changes in both the mucosal and submucosal layers, including neutrophil infiltration and submucosal ulceration (Nakhai et al. 2007).

The use of DPSCs in the present study demonstrated the characteristic morphological features of mesenchymal stem cells, including a fibroblast-like appearance and adherence to growth plates. DPSCs were also characterized by flow cytometry, and their mesenchymal stem cell identity was confirmed through the positive expression of CD90 and CD105 and negative expression of CD34 and CD45. DPSCs were chosen for their ease of accessibility and for their remarkable immunomodulatory properties (Gronthos et al. 2002; Huang et al. 2009b, a; Wada et al. 2013; El-Moataz et al. 2018). In this context, previous studies have reported that MSCs exert regenerative therapeutic effects on induced colitis models by orchestrating and reducing immune cell infiltration into the colon (Zhang et al. 2009). In the present study, compared with those in the other groups, all the investigated macroscopic and microscopic signs of inflammation were markedly improved after the combined administration of sulfasalazine and DPSCs. DPSC transplantation significantly attenuated colonic edema, likely due to the anti-inflammatory characteristics of DPSCs. This effect was further enhanced when DPSCs were combined with sulfasalazine, suggesting that the combination of these agents augmented the anti-inflammatory effect of sulfasalazine, leading to this significant change. Our results align with those of Yousefi-Ahmadipour et al. (2019), who investigated the role of adipose-derived stem cells in combination with sulfasalazine in a rat model of inflammatory bowel disease (Yousefi-Ahmadipour et al. 2019). The dosage utilized in our study was estimated based on prior research conducted on comparable animal models (Yousefi-Ahmadipour et al. 2019). Prior studies have determined that mesenchymal stem cells obtained from bone marrow showed no signs of toxicity at a dosage of 252 × 10^6^ cells/kg b.w. in rats. Thapaswini et al. (2022) reported that DPSCs showed no adverse reactions at concentrations of up to 10 million cells per mL (Thapaswini et al. 2022).

Our histological findings further confirmed the macroscopic results obtained in the validation of the ulcerative colitis model and in the establishment of the healing effect of DPSCs alone or in combination with sulfasalazine. Histopathological examination of colon tissue from acetic acid-induced rats revealed patchy loss of normal surface epithelium, distortion of the normal crypt architecture, degeneration of mucosal cells, and inflammatory infiltration in the mucosa and submucosa in the presence of hemorrhage. This occurrence can be attributed to the impact of acetic acid on the induction of colonic damage, subsequently resulting in epithelial mucosal destruction followed by inflammation. Acetic acid causes inflammation mainly through the infiltration of its soluble protonated form into the epithelium, which in turn causes intracellular acidification, leading to severe destruction of the epithelial layer (Nakhai et al. 2007; Soliman et al. 2016). Sections of the acetic acid- and sulfasalazine-treated group illustrated adequate improvement with minimal reduction of some surface columnar epithelial cells; there was mild inflammatory infiltration in the mucosa and submucosa. These results are in line with the results of previous studies (El-Akabawy and El-Sherif 2019; Yousefi-Ahmadipour et al. 2019). Notably, after DPSC therapy, normal histological structures with intact mucosal epithelium and muscularis layers and only slight inflammatory infiltration in the mucosa and submucosa were observed. These results resemble those of Yousefi-Ahmadipour et al. and could be explained by the possibility that the engrafted DPSCs differentiated into colonic interstitial lineage cells (Yousefi-Ahmadipour et al. 2019). However, the combined administration of DPSCs and sulfasalazine resulted in a noticeable improvement in histological structure, with an intact mucosal epithelium and mild inflammatory infiltration in the mucosa and submucosa, with slight hemorrhage.

To further elaborate the potential therapeutic role of DPSCs in the treatment of ulcerative colitis, oxidative stress markers (ROS and XOD) were investigated. Oxidative stress is a crucial factor in the pathogenesis of ulcerative colitis progression. The ROS concentration reflects the degree of oxidative stress associated with inflammation due to the occurrence of a certain amount of damage. On the other hand, XOD functions as an antioxidant that degrades ROS and prevents cells from undergoing peroxidation. The release of inflammatory cytokines disrupts the equilibrium between antioxidation and oxidation, which triggers the inflammatory response. This in turn causes damage to intestinal cells and destruction of the mucosal barrier (Wang et al. 2019; Benhar 2020). In our study, the administration of either DPSCs or sulfasalazine, either individually or in combination, significantly reduced ROS levels and significantly increased XOD activity. The most prominent and significant improvement was observed when sulfasalazine and DPSCs were administered in combination. The inhibitory effect of DPSCs on oxidative stress has been documented by (Al-Serwi et al. 2021) in a rat model of diabetes mellitus. Mesenchymal stem cells (MSCs) have both enzymatic and nonenzymatic biological mechanisms that help in neutralizing ROS (Hernández-Monjaraz et al. 2020). Moreover, DPSCs have been found to possess antioxidative characteristics (Song et al. 2015), supporting the proposed antioxidant abilities of MSCs (Silva et al. 2003).

Regarding sulfasalazine, Joshi et al. (2005) studied the reactions of sulfasalazine and its metabolites with various oxidizing and reducing free radicals to understand the mechanistic aspects of sulfasalazine action against free radicals produced during inflammation. Those investigators emphasized the ability of sulfasalazine and its metabolites to scavenge various primary and secondary free radicals. In addition, a recent study by Liu et al. (2020) revealed that sulfasalazine attenuates acetic acid-induced colitis in rats via the inhibition of oxidative stress (Liu et al. 2020). Furthermore, the antioxidative mechanism of sulfasalazine as a major path of pharmacological action against colitis has been confirmed by the latest study of Abdelmonaem et al. (2021), who found that sulfasalazine administration in colitis-bearing rats significantly reduces the colonic level of malondialdehyde, an indicator of oxidative stress, and normalizes total antioxidant capacity and superoxide dismutase activity, which are indices of the antioxidant defense system (Abdelmonaem et al. 2021).

As expected, acetic acid administration significantly elevated the levels of all the studied proinflammatory cytokines (TNF-α, NFκB-P65, and TLR-4) in the colon. Although the administration of DPSCs or sulfasalazine alone led to a significant reduction in the levels of the tested proinflammatory cytokines in the colon, the combination of both DPSCs and sulfasalazine drastically decreased the levels of those cytokines in the colon. Consequently, we proposed that the combination of DPSCs and sulfasalazine ameliorates UC through the inhibition of both inflammatory and oxidative stress processes.

Furthermore, the immunohistochemical results of the present study demonstrated that the combined administration of DPSCs and sulfasalazine attenuated NFκB-p65 and IL-1β expression. Interleukin-1β (IL-1β) is a major cytokine associated with the activation of proinflammatory signaling pathways and monocytes in peripheral tissues and has been described as an important mediator of the onset of ulcerative colitis-related inflammation (Liso et al. 2022). On the other hand, NF-κB was reported to play a crucial role in the pathogenesis of ulcerative colitis, as indicated by the upregulation of the expression of several inflammatory markers (Li et al. 2005). Interestingly, neither DPSCs nor sulfasalazine were able to reduce IL-1β and NFκB-p65 expression on their own. These findings provide further evidence for the hypothesis that combined therapy involving both DPSCs and sulfasalazine is fundamental for optimizing the efficacy of both agents, which will in turn contribute to more beneficial clinical results.

Finally, we further investigated the molecular role of both DPSCs and sulfasalazine through analyzing the gene expression levels of MyD88, NF-κB and TLR4 in an ulcerative colitis-induced rat model. MyD88 plays a pivotal role in modulating the immune response and inflammatory processes associated with colitis (Saikh 2021). It has been reported that NF-KB activation in patients with inflammatory bowel disease promotes mucosal inflammation by enhancing the expression of numerous proinflammatory genes (Atreya et al. 2008; Mohammad Jafari et al. 2021). Like the pattern delineated in the results previously outlined in the present study, the combined administration of DPSCs and sulfasalazine significantly downregulated MyD88, NF-κB and TLR4 gene expression. The specific mechanism underlying the anti-inflammatory and immunomodulatory activities of DPSCs is complicated. DPSCs likely modulate immune cells via inflammatory immune-related signaling pathways (Li et al. 2023). DPSCs can regulate different types of immune cells and consequently act as effective immunomodulators by suppressing proinflammatory processes and promoting anti-inflammatory processes (Földes et al. 2016; Li et al. 2023). A study conducted by Folds et al. (2016) demonstrated that intravenous administration of human DPSCs can effectively alter the progression of mouse acute colitis induced by dextran sodium sulfate (DSS). These findings confirmed the anti-inflammatory function of DPSCs in the treatment of inflammation in experimental colitis (Földes et al. 2016). Additionally, Zhao et al. (2012) established that systemic infusion of DPSCs in mice suffering from DSS-induced colitis significantly reduced inflammatory cell infiltration and improved overall signs of colitis (Zhao et al. 2012). The processes related to the anti-inflammatory and immunomodulatory effects of DPSCs depend on the suppression of activated T-cell proliferation (Sonoyama et al. 2008) and the modulation of both B cells and NK cells, hence restricting adverse inflammatory and immunological reactions (Gerdoni et al. 2007). DPSCs play a vital role in the dynamics of inflammation through their capacity to modulate the immune system. This effect is achieved by direct interaction between cells and the release of chemicals that mediate the inflammatory response (Albashari et al. 2020). Bousnaki et al. (2022) employed proteomics to investigate the anti-inflammatory effects of the DPSC secretome under various circumstances (Bousnaki et al. 2022). These findings suggest that DPSCs have tremendous potential as an effective treatment for inflammatory diseases. Regarding the anti-inflammatory role of sulfasalazine, a previous report by Abdelmonaem et al. (2021) confirmed the anti-inflammatory role of sulfasalazine in a similar model of ulcerative colitis, as indicated by the significant inhibition of serum IL-1β and colonic Il-18 levels (Abdelmonaem et al. 2021). Several prior reports have suggested that the anti-inflammatory effects of sulfasalazine include the suppression of inflammatory cell chemotaxis (Molin and Stendahl 2009), the inhibition of lymphocyte proliferation and activation (Imai et al. 1991), and the suppression of antibody production and free radical scavenging. Gan et al. proposed that the mechanism of action of sulfasalazine in ulcerative colitis depends on the inhibition of NF-κB activation (Gan et al. 2005). The regulation of proinflammatory cytokines by the transcription factor NF-κB is well established, and elevated expression of these cytokines has been linked to the onset and persistence of intestinal inflammation in patients with inflammatory bowel diseases (Atreya et al. 2008). Hence, the role of sulfasalazine in the regulation of NF-κB and the control of the inflammatory response may be beneficial for the treatment of UC. Hence, our results indicated that the reported therapeutic advantages achieved through the combination of DPSCs, and sulfasalazine may be attributed to their synergistic effect in downregulating the generation of proinflammatory cytokines, suppressing various inflammatory pathways, such as the NF-κB pathway, and regulating oxidative stress.

## Conclusion

In light of the present findings, it could be concluded that the use of DPSCs in combination with sulfasalazine effectively enhanced the treatment outcome in a rat model of UC induced by acetic acid. This could be attributed to the combined impact of these two factors on ameliorating the damaging insult caused by these debilitating conditions. The mechanism involved in this effect is closely related to the synergetic effects of both DPSCs and sulfasalazine on the suppression of the inflammatory response and the regulation of oxidative stress. However, importantly, our experimental study faced certain limitations. Initially, the inflammation induced by the administration of acetic acid, which mostly resembles ulcerative colitis, was not completely comparable. The primary cause of ulcerative colitis is immunological activation, while acetic acid leads to severe damage to the mucosal lining. Therefore, additional research should be conducted on other models. Furthermore, it is advisable to consider the long-term implications of the combined use of DPSCs and sulfasalazine in the treatment of this chronic disease. Considering the aforementioned factors, the findings reported in this study could prove to be significant in the development of a cell-based strategy for the treatment of UC, either independently or in combination with conventional medications.

## Data Availability

The datasets generated during and/or analyzed during the current study are available from the corresponding author upon reasonable request.

## Abbreviations

UC: Ulcerative colitis
IBD: Inflammatory bowel disease
DPSCs: Dental pulp stem cells
MSCs: Mesenchymal stem cells
DMEM: Dulbecco’s modified Eagle’s medium
FBS: Fetal bovine serum
PBS: Phosphate-buffered saline
FACS: Flow cytometric analysis
ELISAs: Enzyme-linked immunosorbent assays
NF-κB-P65: Nuclear factor kappa B
TNF-α: Tumor necrosis factor alpha
TLR-4: Toll-like receptor 4
